# Insights into mechanisms of seed longevity in soybean: a review

**DOI:** 10.3389/fpls.2023.1206318

**Published:** 2023-07-21

**Authors:** Polneni Jagan Mohan Rao, Mandalapu Pallavi, Yarasi Bharathi, P. Bindu Priya, Patta Sujatha, Kona Prabhavathi

**Affiliations:** Seed Research and Technology Centre, Professor Jayashankar Telangana State Agricultural University, Rajendranagar, Hyderabad, India

**Keywords:** soybean, seed longevity, genetic, molecular, physiological, biochemical

## Abstract

Soybean, a crop of international importance, is challenged with the problem of seed longevity mainly due to its genetic composition and associated environmental cues. Soybean’s fragile seed coat coupled with poor DNA integrity, ribosomal dysfunction, lipid peroxidation and poor antioxidant system constitute the rationale for fast deterioration. Variability among the genotypes for sensitivity to field weathering contributed to their differential seed longevity. Proportion and density of seed coat, glassy state of cells, calcium and lignin content, pore number, space between seed coat and cotyledon are some seed related traits that are strongly correlated to longevity. Further, efficient antioxidant system, surplus protective proteins, effective nucleotide and protein repair systems and free radical scavenging mechanisms also contributed to the storage potential of soybean seeds. Identification of molecular markers and QTLs associated with these mechanisms will pave way for enhanced selection efficiency for seed longevity in soybean breeding programs. This review reflects on the morphological, biochemical and molecular bases of seed longevity along with pointers on harvest, processing and storage strategies for extending vigour and viability in soybean.

## 1 Introduction

Soybean (*Glycine max* L. Merill) is an important oilseed crop contributing to 30 per cent of the world’s edible oilseed production. Owing to its superior nutritional qualities, soybean is considered as a miracle crop and is cultivated in many developing countries to meet their growing demand for protein and oil (Khurshid et al., 2017). Seed of soybean is a powerhouse of nutrients consisting of 40 to 45% protein, 20 to 22% oil and 20 to 26% carbohydrate besides high amounts of calcium, phosphorous and vitamins (Rahman et al., 2011). Globally it is cultivated in an area of 124.92 m ha with production and productivity of 348.71 mt and 2791 kg ha^-1^ respectively (INDIASTAT, 2018). India ranks fifth among major soybean growing countries in the world with 12.92 m ha of cultivated area producing12.61mt at a productivity of 976 kg ha^-1^ (INDIASTAT, 2020-21).

Both production of highly vigorous seed and retention of seed vigour are pivotal for profitable seed production. Short life span of soybean seed (< 8 months in tropics) under ambient storage conditions forbids its usage in the ensuing seasons by farmer thus burdening the seed industry with entire seed supply. It is therefore pertinent to develop a complete seed supply chain for soybean duly maintaining its quality at all stages beginning from harvest till sowing. Thus seed longevity assumes a key role in preserving the seed fitness during storage under ambient conditions. However, maintaining seed longevity is challenging in soybean given its inherent shorter life span besides meager understanding of the mechanisms regulating seed longevity during late maturation (Shelar et al., 2008). Poor seed longevity leads to unexpected losses in seed viability during storage, negatively impacts seedling establishment and ultimately affects crop yield (Walters, 1998; Finch-Savage and Bassel, 2016).

Seed longevity is influenced by the ability to stabilize the biological entity for longer period by formation of an amorphous, highly viscous, solid-like matrix (*i.e.*, a glassy state) in cells that suspends integrated metabolic activities and severely slows down deteriorative reactions (Walters, 1998; Buitink et al., 2000; Walters et al., 2010). Seed longevity is also attributed to a range of protective compounds (Sano et al., 2016; Leprince et al., 2017) including non-reducing soluble sugars (sucrose and raffinose family oligosaccharides-RFOs) (Salvi et al., 2016; Zinsmeister et al., 2016) and a set of late embryogenesis abundant (LEA) proteins and heat shock proteins (HSP) (Tejedor-Cano et al., 2010; Hundertmark et al., 2011; Chatelain et al., 2012). Longevity is also conferred by antioxidants that limit oxidation of lipids, proteins and nucleic acids during storage such as glutathione (Nagel et al., 2015), tocopherols (Debeaujon et al., 2000; Sattler et al., 2004), flavonoids present in the seed coat (Debeaujon et al., 2000) and lipocalins (Boca et al., 2014). Several repair mechanisms also contribute to longevity when they are activated during seed imbibition to fix damage that occurred to proteins and DNA during storage (Oge et al., 2008; Waterworth et al., 2010). In addition to protection and repair, an impaired degradation of chlorophyll appears to negatively influence longevity (Nakajima et al., 2012; Zinsmeister et al., 2016). Presence of chlorophyll is considered as an indicator of immaturity but how it affects longevity remains unsolved.

Seed ageing is an inexorable and irreversible process influenced by both intrinsic and extrinsic factors and detected by cytological, physiological, biochemical and physical changes associated with decline in seed quality and viability (Nadarajan et al., 2023). Seed deterioration is considered to begin at physiological maturity (*ie*., pre-harvest) and continue during harvesting, processing and storage at a rate greatly influenced by genetic, production and environmental factors (Hampton and Tekrony, 1995). Thus high germinating seed lots that are chronologically of same age may deteriorate at different rates, henceforth differing markedly in seed vigour. The rate of deterioration fluctuates critically from one species to another and also among the same species depending on the crop growth conditions and genetic lineage (Chau et al., 2019 and Crane et al., 2003). Deterioration is evident as a reduction in percentage germination and field emergence, increased number of weak/abnormal seedlings, loss of vigor and viability ultimately causing seed death (Maity et al., 2000; Tilebeni and Golpayegani, 2011). The internal factors which contribute to the process of seed deterioration are loss of membrane integrity, change in the structure of macromolecules (Barton, 1961; Wilson and Mc Donald, 1986; Wettlaufer and Leopold, 1991), enzyme degradation (Barton, 1961; Bailly et al., 1998; Ravikumar et al., 1998), impairment of RNA and protein synthesis and DNA degradation. The deteriorative alterations occurring with time along with exposure to external challenges decreases the ability of seed to survive as it is accompanied with loss of quality, viability and vigour. The rate of deterioration rapidly surges with increase in either seed moisture content or storage temperature (Ellis et al., 1991).

Speed of deterioration depends on the storage environment and also particularities of the species like the seed chemical composition. In soybean, linoleic acid content in seed is largely influenced by the intensity of lipid peroxidation rather than its oil and oleic acid contents (Tatić et al., 2012). Oilseeds are highly sensitive to storage environment and lose viability at a quicker pace due to high fatty acids and fragile seed coats. Fatty acid composition is one of the key factors determining the susceptibility of oils to oxidation process (Shaban, 2013). It limits seed longevity due to decline in oil content and germination percent during storage. Lipid auto oxidation and changes in fatty acid content during storage are the most often mentioned reasons for accelerated damage of seed in oilseed crops (Tatić et al., 2012). The enzyme lipase, abundantly produced during storage, breaks down lipids into free fatty acids and glycerol. Accumulation of reactive oxygen species and free radicals is considered as one of the important elements of seed ageing. Though an irreversible process, seed ageing can be swayed with anti-oxidants like α- tocopherol, ascorbic acid, glutathione carotenoids and phenolic compounds such as flavonoids that offer protection from free radicals or produce halogen vapours to stabilize unsaturated fatty acids thereby reducing the amount of oxygen around the seed and decreasing the initiation of free radicals. In view of the accruing international importance of soybean and yet the unsolved hitches, this review frames out the major causes and challenges pertaining to seed longevity along with conceptualizing heuristic strategies for prolonged longevity of soybean seed.

## 2 Mechanisms of seed deterioration

### 2.1 Cellular degradation

#### 2.1.1 Membrane damage

**Figure 1:**
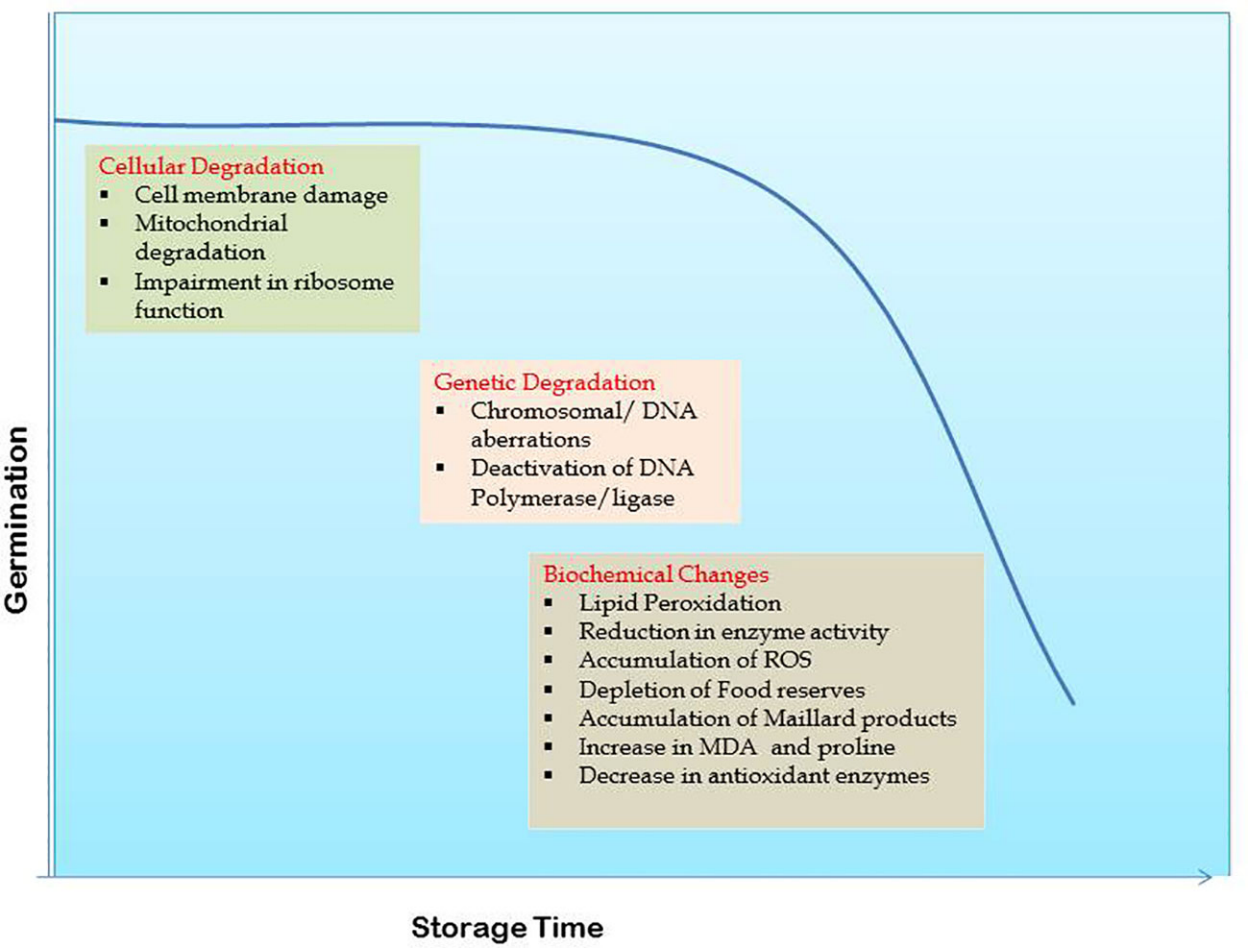
Seed deterioration mechanism in soybean.

Cell membrane deterioration (Berjak and Villiers, 1972; Priestley and Leopold, 1986; Ferguson et al., 1990; Aiazzi et al., 1997) and damage to nucleic acids (Chen et al., 2013; Fleming et al., 2019) are the two major aspects of seed deterioration during storage (**Figure 1**). Cellular deterioration begins with membrane damage which on losing permeability allows leaching of cytoplasmic contents into the intercellular spaces. Membrane disintegration is triggered by the hydrolysis of phospholipids and their auto oxidation. Increased solute leakage, higher free fatty acid levels, loss of membrane phospho lipids, lower antioxidant potential of lipids, lower tocopherol content and reduced ascorbate-dehydroxyascorbate ratio indicative of oxidative stress are the key age-induced changes observed in soybean seed (Senaratna et al., 1988). Loss of seed viability is correlated to phospholipase D activity that is accounted for detachment of plasma membrane from cell wall complex and disorganization of oil bodies during natural ageing in soybean (Lee et al., 2012). Membrane permeability in seeds increases with age causing a surge in electrical conductivity (EC) (Ferguson et al., 1990).

#### 2.1.2 Mitochondrial degradation

Loss of membrane integrity disturbs the functionalities of all membrane bound organelles including mitochondria. Seed vigor declines in parallel to cell turgor in seeds with disturbed membrane integrity (Parrish and Leopold, 1978) (**Figure 1**). Seed ageing is closely linked with mitochondrial degradation and associated functional changes such as loss of its natural swelling and contracting ability causing to permanently remain in swollen state, followed by pigmentation and fragmentation. Inflated ATPase and depressed ability of oxidative phosphorylation are key aspects of mitochondrial deterioration (Priestly, 1986; Walters, 1998; Murthy et al., 2002). Mitochondrial membranes comprising of phospholipids and proteins, and specifically the inner one has a strong influence on seed viability when disorganized (Tatipata, 2009). With ageing, inner membrane vesiculates, cristae disappear and ATP synthase dissociates leading to impaired supply of ATP to cell by mitochondria (Daum et al., 2013). Further, activities of mitochondrial antioxidant enzymes significantly drop in aged seeds. There is down regulation of cytochrome oxidase pathway, tricarboxylic acid cycle, mitochondrial protein levels and many other associated proteins (Yin et al., 2016). Retarded mitochondrial and ascorbic acid-glutathione cycle activities along with increased reactive oxygen species (ROS) accumulation cause mitochondrial dysfunction in aged soybean seeds (Xin et al., 2014). Mitochondria with accumulated excessive ROS negatively impact the antioxidant system activity leading to seed deterioration (Kurek et al., 2019).

#### 2.1.3 Ribosomal dysfunctioning

Impairment of ribosome function is manifested as non-dissociation of polyribosomes before attachment of preformed mRNA affecting the process of protein synthesis. In non-viable seeds the ribosomes fail to dissociate and protein synthesis is retarded which is a measurable symptom of ageing (Smith, 1995 and Walters, 1998).

### 2.2 Genetic degradation

In several plants, chromosomal or DNA aberrations within cells have been observed along with loss of seed viability (Abdalla and Roberts, 1968). Fragmentation of embryonic nuclear DNA and its repair processes progress slowly during ageing due to deactivation of certain key enzymes like DNA polymerase and DNA ligase (Coello and Vazquez-Ramos, 1996; Schoen et al., 1998; Chwedorzewska et al., 2002) (**Figure 1**). Potential targets for oxidative damage in the DNA chain include purine and pyrimidine bases and deoxyribose sugars (Larson, 1997; Roldan-Arjona and Ariza, 2008). Volatile aldehydes formed from lipid peroxidation cross link with macromolecules like sugars, amino acids or the polypeptide chain, altering their structure and increasing the occurrence of genetic mutations during seed ageing. Polymerase chain reaction (PCR) using primers, has been widely used for studying these genetic changes. The RAPD profiles of DNA from differently aged soybean seeds showed polymorphism (Shatters et al., 1994) while natural deterioration had no effect on RAPD markers in seeds stored for longer periods (Zhang et al., 1996). Later on, a set of thirteen selected SSR loci for DNA profiling of soybean cultivars was identified (Song et al., 1999). Such studies assist in taking a deeper look into the effects of seed ageing through molecular markers and DNA fingerprinting.

### 2.3 Biochemical changes

#### 2.3.1 Lipid peroxidation

Seed viability is directly influenced by membrane lipid functioning though exactly how it is affected is yet to be fully unraveled. Most of the oilseed plants are highly prone to lipid peroxidation induced damages. Lipid peroxidation is an oxidative damage affecting cellular membranes, lipoproteins and other molecules that contain lipids (**Figure 1**). The extent of lipid peroxidation is greatly influenced by the seed moisture content and storage temperature (Murthy et al., 2002). Recent studies suggest that catalysis of lipid hydrolysis mediated by lipases occurs even in dry seed as the microenvironment of oil bodies permits diffusion of the enzyme (Wiebach et al., 2020). Lipid peroxidation is a chain of reactions initiated by free radicals resulting from stress and influencing the unsaturated fatty acids in cell membranes leading to their damage. These free radicals act as both initiators and terminators of lipid peroxidation process and once activated, these reactions continue auto catalytically causing structural and functional changes to the substrate (Ognjanovic et al., 2008). Enzymatic antioxidants like superoxide dismutase (SOD), catalases and peroxidases, and non-enzymatic antioxidants like ascorbic acid and glutathione control the levels of ROS wherein ascorbic acid content and activity typically decrease during seed maturation while glutathione plays a key role as redox buffer (Nadarajan et al., 2023). Thus reduced non-enzymatic antioxidant contents and catalase activity causes accumulation of ROS eventually leading to lipid peroxidation in soybean (Lin et al., 2022). The down-regulation and reduction in scavenging antioxidant activity is associated with depression of the antioxidant enzyme (Yin et al., 2016). This imbalance of the antioxidant system leads to the accumulation of reactive oxygen species (ROS), especially hydrogen peroxide (H_2_O_2_) due to its relatively long half-life (1 ms) compared to the other forms of ROS (2–4 µs) (Gill and Tuteja, 2010). Moreover, lipoxygenase, an oxidative enzyme present in many unimbibed seeds is also capable of producing activated oxygen and subsequently catalyzing lipid peroxidation by using membrane and phospholipid components as substrates (Priestly, 1986). Enzyme phospholipase D catalyzes phospholipid hydrolysis causing compositional changes in triacylglycerols (storage lipids) and its suppression can increase seed longevity in soybean (Lee et al., 2012). In *Jatropha curcas*, however, seed deterioration is correlated to changes in the antioxidant enzymatic activities of embryo with no relation between deterioration of seed during storage and lipid peroxidation activity (Silva et al., 2017).

Changes in the chemical constituents of cell also correlate with seed viability thus serving as indirect measures of seed longevity like rise in electrical conductivity of seed leachates associated directly with deterioration in soybean (Srivastava and Gill, 1975). On the contrary, when seeds were stored at 10°C for 18 months, bulk electrical conductivity test could not establish an association between fatty acids and carbohydrate changes and hence cannot be considered as an useful indicator of seed degradation (Panobianco and Vieira, 2007). Accumulation of volatile aldehydes is another indicator of lipid peroxidation and thereto seed deterioration. These volatile aldehydes are the secondary products of lipid peroxidation that dehydrate fatty acids to smaller volatile carbon compounds like hexanals, pentanals and butanals. Stored soybean seeds showed decline in protein, lipid and poly unsaturated fatty acid contents and increase in hexanals (Braccini et al., 2000).

#### 2.3.2 Enzymatic changes

Analysis of enzymatic changes is yet another tool to understand seed deterioration during ageing. The most prominent hypothesis regarding seed ageing points towards the structural changes at macromolecular level leading to enzymatic degradation (**Figure 1**). A positive correlation exists between the enzyme’s antioxidant capacity and seed vigour while lessened antioxidant enzymatic activity during ageing (Ebone et al., 2020) is analogous to enhanced lipid peroxidation (Bailly et al., 1998). Prolonged storage causes rapid reduction in the activities of superoxide dismutase, α-amylase, dehydrogenase, catalase and ascorbate peroxidase enzymes (Bailly, 2004; Balešević-Tubić et al., 2011; Yadollhhi and Mashayekhi, 2013; Radha et al., 2014; Pawar et al., 2017; Brar et al., 2019; Das and Biswas, 2022). This leads to higher accumulation of free radicals and weakened seed viability. Likewise, decline in esterase and glutamate dehydrogenase activities leads to low seed vigour under varying storage periods (Vieira et al., 2013). High seed moisture content (upto levels necessary for germination) activates some hydrolytic enzymes that stimulate seed ageing and any further increase in moisture content leads to speedy deterioration of seed because of energy expenditure and accumulation of breakdown products (Copeland and McDonald, 1995). Under low storage moisture conditions, inflation of toxic compounds that reduce seed viability occurs due to reduced respiration and enzyme activity. During accelerated ageing, lipid peroxidation causes loss of free radical scavengers resulting in seed deterioration (Bailly et al., 1998). Soybean genotypes with higher longevity recorded greater lipoxygenase II and antioxidative enzymatic activities that lessened lipid peroxidation and improved viability throughout storage (Vijayakumar et al., 2019).

#### 2.3.3 Metabolic changes

Decline in germination ability with ageing is manifested due to depletion of total sugars, protein and oil contents in seed (Mahjabin et al., 2015) (**Figure 1**). A complete proteomic analysis of soybean seed subjected to controlled deterioration revealed degradation of proteins involved in primary metabolism and energy metabolism leading to an impairment of ATP synthesis and also decreased modulation of protein synthesis related proteins. These modulations in *de novo* protein synthesis are one of the major contributors for decreased seed longevity (Min et al., 2017). The role of chemical protein reactions in loss of seed viability was investigated through studies on Maillard products. This reaction is catalysed by glycosylation or glycation associated with the covalent attachment of reducing sugars to amine groups of amino acid and protein to form glycated protein. Decrease in seed germination along with accumulation of Maillard products in soybean embryos could be attributed to the role of Maillard reaction (Wettlaufer and Leopold, 1991). A novel non-destructive method of measuring the respiratory products in seeds was associated with changes in seed metabolic activity apart from decrease in viability and quality with ageing (Kranner and Gill, 1996). The naturally ageing soybean seed in glassy state inhibits the formation of reducing sugars from hydrolysis of oligosaccharides delaying the ageing process (Sun and Leopold, 1995). Fall in carbohydrate and protein contents (Verma and Ram, 1987), increased MDA and proline contents and low concentrations of antioxidant enzymes (Abdulla and Farah, 2014) in ageing seeds emphasize lipid peroxidation activity. In general, seed stored for longer periods shows decreased metabolic activity and lower potential to produce nucleic acids and nucleotides (Mahjabin and Abidi, 2015).

## 3 Factors influencing seed longevity in soybean

Seed, the basic input of agriculture, is highly sensitive to any sort of damage, even more so in case of soybean. The environment prevailing during seed production, development and maturation has great influence on seed longevity and vigour.

### 3.1 Intrinsic factors

Germination ability and seed vigour are maximum at physiological maturity *i.e.*, when seed attains maximum dry weight. Radicle hypocotyl, the most crucial part for germination is vulnerable to damage during harvesting and processing in soybean because of its position and delicate seed coat (Kuchlan, 2006). Longevity is developed during seed filling to seed maturation stages (Zanakis et al., 1994; Verdier et al., 2013; Leprince et al., 2017) though some opine that highest longevity is acquired during seed filling stage (Gillen et al., 2012; Filho, 2016). Longevity is maximum at peak physiological maturity after acquisition of desiccation tolerance and shortly before the end of seed filling and onset of maturation drying (Lima et al., 2017). Thereafter, the time required to achieve 50% germination (P50) increased by two times from further maturation to stage R9 *ie*., dry mature seeds. There is increasing evidence on synthesis of protective mechanisms that enhance longevity being induced sequentially and increase progressively during late seed maturation (Probert et al., 2007; Righetti et al., 2015; Leprince et al., 2017). Soybean seeds harvested at different developmental stages show varied levels of protective compounds and thereto varied longevity periods.

Hilum is another prime seed part that delivers nutrients and photosynthates to the developing embryo and when damaged leads to seed infections and inferior seed quality. Likewise, properties of seed coat surface like cutin deposition, cuticle cracks, gap between seed coat and cotyledon and depositions influence water permeability, fungal invasion, and ultimately seed longevity in soybean (Ranathunge et al., 2010; Kuchlan et al., 2018).

### 3.2 Extrinsic factors

In addition to the internal aspects, environmental stresses during seed development and maturation phases also impair seed longevity. Continuous precipitation after crop maturation and prior to harvest leads to wetting and drying of seed resulting in seed deterioration. Hot and dry weather during harvest adversely affects both physical and physiological qualities of soybean seed (Green et al., 1971). Similarly water stress during seed development results in mature green seeds with poor seed longevity (Smolikova and Medvedev, 2016). The maturation environment (*i.e*., humidity and temperature) fluctuations influence seed vigour as they not only accelerate respiration and consume the food reserves essential for seedling development, but also trigger the formation of toxic compounds which degrade plasmatic membranes of the seeds (Aumonde et al., 2017). A stimulated rainfall of 120 and 180 mm at R8 stage of soybean promotes significant reduction of germination and seed vigour in comparison to 0 and 60 mm along with reduction in antioxidative enzyme activity, accumulation of hydrogen peroxide and malondialdehyde, protein content and protease activity (Pinheiro et al., 2023). Water deficiency during different stages of plant growth results in reduced seed development due to decrease in photosynthesis, assimilation and translocation to developing seeds (Sobko et al., 2020). High (>35°C) as well as low (<15-17°C) temperatures result in reduced seed filling and retarded seed growth respectively (Liu et al., 2008). Higher water availability throughout the vegetative growth phase results in delayed flowering with enhanced assimilation during seed development (Kuswantoro, 2018). Apart from temperature, relative humidity also greatly influences seed longevity, so the seeds should be stored as per Harrington’s principles.

Mechanical damage during harvesting and threshing is a common problem encountered in soybean. The seed becomes brittle and easily prone to mechanical injuries when moisture content drops below 12 percent (Delouche and Gill, 1973). It is therefore suggested to harvest the crop at an optimum seed moisture content of 13 to 15 percent (Park and Webb, 1959; Popinigis, 1972). It is also recommended to store soybean at reduced oxygen levels *i.e.*, below 0.77MPa oxygen pressure to extend seed longevity (Ohlrogge and Kernan, 1982).

## 4 Reinforcing systems of seed longevity in soybean

### 4.1 Protective systems

#### 4.1.1 Seed and seed coat components

**Figure 2:**
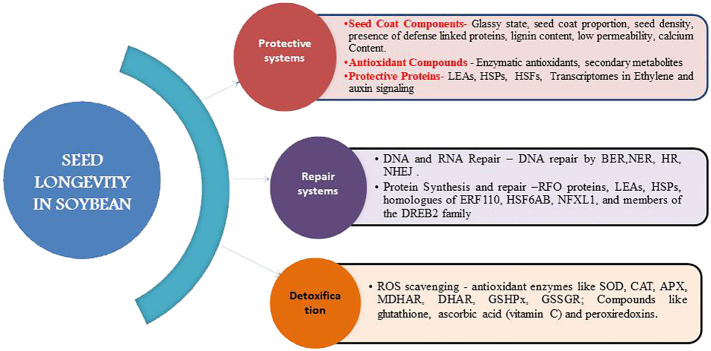
Reinforcing systems of seed longevity in soybean.

Seed coat is an important protective agent that shields the underlying embryo from biotic and abiotic stresses (**Figure 2**). The degree of protection offered relates to the structural arrangement and composition of seed coat. Several defense-linked proteins such as polyphenol oxidases (eg., catechol oxidases and laccases), peroxidases and chitinases are prevalent in the seed coat testa (Moïse et al., 2005; Pourcel et al., 2005). The lignin content of soybean testa also correlates with seed permeability and resistance to mechanical damage (Debeaujon et al., 2007). Generally high seed permeability is associated with rapid deterioration while seed coat lignin content is negatively correlated with seed permeability (Abati et al., 2022). A decrease in seed coat deficiency in natto soybean varieties is associated with alleles governing higher lignin biosynthesis (Zhu et al., 2020). The cell wall organization, callose deposition in cell wall, cuticle development, cell wall modification, and secondary cell wall biogenesis are governed by Glyma.15G078300, Glyma.15G075300, Glyma.15G074700, Glyma.15G074000, and Glyma.15G072300 group of genes (Shao et al., 2007). The rate of oxygen consumption estimated through Q2 seed analyser is associated with germination capacity of seed and is affected by seed coat permeability and seed size besides dormancy status and imbibition rate (Nadarajan et al., 2023). Black seeded soybean with few pores on seed coat, high lignin content and narrow space between seed coat and cotyledon possesses greater longevity (Kuchlan et al., 2010). Seed coat impermeability is also associated with the presence of phenolic (Chachalis and Smith, 2001; Zhou et al., 2010) and more importantly epicatechin type of phenolic compounds that positively influence the hard seed percentage (Zhou et al., 2019). The permeability of wild soybean can be linked to the free deposit with larger cuticle cracks on seed coat (Vu et al., 2014). Calcium content of seed coat is another factor accountable for hard-seededness and therefore low germination with prolonged longevity as depicted in seed coats of GmHs1-1 transgenic soybean lines that had more calcium than wild type (Sun et al., 2015). Two black seeded genotypes ACC No.369 and ACC No.39 reported high seed longevity both under natural and accelerated ageing conditions (Naflath and Ravikumar, 2023). Therefore small sized black coated soybeans with high lignin and calcium contents, higher secondary metabolites and lower seed coat permeability effect prolonged seed longevity in soybean.

Seed coat proportion is yet another essential feature impacting the protection offered against mechanical damage during harvesting and processing. Proportion of seed coat has a positive association with seed longevity as evident from higher proportion of seed coat in the black-seeded varieties compared to yellow-seeded ones (Kuchlan et al., 2010). Seed density also associates positively with seed longevity (Adsul et al., 2018). Likewise, seed longevity can also be attributed to development of glassy state of the cells which halts the integrated metabolic activities thus slowing down the deteriorative reactions by curtailing the production of toxic compounds (Buitink et al., 2000; Walters et al., 2005; Walters et al., 2010). Rapid decrease in the seed viability of accelerated aged soybean seeds is associated with loss of glassy state (Sun and Leopold, 1994) during which lipid peroxidation and Maillard reactions are prevented (Karmas et al., 1992).

#### 4.1.2 Antioxidant compounds

Accumulation of antioxidant components in dry seeds during late maturation phase on mother plant contributes to storability potential. Enzymatic antioxidants comprising ascorbate peroxidase, superoxide dismutase (SOD), glutathione reductase, catalase, peroxidase, glutathione peroxidase, *etc*., play a key role in scavenging the accumulated free radicals especially SOD providing the first line of defense against ROS (Bailly et al., 1996; Kuchlan, 2006). Likewise, the protective role of antioxidant secondary metabolites such as flavonoids, vitamin E (tocopherols and tocotrienols), ascorbic acid and glutathione carotenoids during aging or oxidative stress is well documented (**Figure 2**). Poor storability in soybean is due to high hydroperoxidelyase activity during storage causing excess release of volatile aldehydes (Hosamani et al., 2013). Furthermore, it is also regarded that the cause for low storability in aged soybean seeds is not lack of energy reserves, but lack of capability to catabolize the available energy reserves to sucrose (Zhou et al., 2020). Thus seed longevity is a function of both energy storage and its metabolism. In addition, seed treatment with RNS (Reactive Nitrogen Species) compounds like NO or NO donors prior to or during initial stages of aging activates antioxidant systems and triggers defense mechanisms leading to delayed aging (Ciacka et al., 2020).

#### 4.1.3 Protective proteins

The LEAs, HSPs and seed storage proteins are vital for desiccation tolerance and maintenance of a quiescent state in orthodox seeds (**Figure 2**). They also aid in trapping the ROS and protecting cellular structures and other seed proteins from oxidative stress. Furthermore, they help in stabilization of glassy cytoplasm, protection of structural proteins, condensation of chromatin and dismantling of thylakoids in chloroplasts (Sano et al., 2015; Ballesteros et al., 2020) Increased number of transcripts encoding HSPs and heat shock factors (HSFs) offer resistance against accelerated ageing in soybean (Lima et al., 2017). Defensive proteins like polyphenol oxidases, peroxidases and chitinases playing a protective role, accumulate in soybean seed testa during stress (Ranganathan and Groot, 2023). Protein content of soybean seed from all nodal positions decreased vastly after 180 days of storage leading to deterioration in longevity (Sharma et al., 2013). Up-regulation of various HSPs identified as differentially expressed genes (DEGs) was accompanied with high temperature and humidity except Glyma.15G088000 which is found in susceptible soybean cultivars under no stress conditions (Shu et al., 2019). Also, the transcriptomes associated with seed longevity during maturation contain several transcription factors involved in ethylene and auxin signaling (Lima et al., 2017). Soybean seed storage proteins (SSPs) accumulated during seed development largely account for its commercial and nutritional value (Dean and Finer, 2023).

### 4.2 Repair systems

#### 4.2.1 DNA and RNA repair

Seeds are subject to DNA lesions, not only during desiccation but also during seed storage (Osborne et al., 1980). Seed repair mechanisms like priming help in DNA repair through repair pathways like base excision repair (BER) where single strand breaks (SSBs) are repaired by nucleotide excision repair (NER) and double strand breaks (DSBs) by homologous repair (HR) or non-homologous end joining (NHEJ) mechanisms (**Figure 2**). For the same reasons, their induction during seed invigoration treatments is of special interest to the seed industry.

RNA being single-stranded, is most vulnerable to damage. The ABA-mediated transcription plays a vital role in determining stored mRNA profiles (Kimura and Nambara, 2010). Quantity of mRNA also increases from the stress tolerance response genes and proteins like LEAs and HSPs. Small non-coding RNAs (miRNAs and siRNAs) though do not code for any protein, have a key role to play in DSB repair mechanism (Rzeszutek and Betlej, 2020).

#### 4.2.2 Protein synthesis and repair

The protective compounds (Sano et al., 2016; Leprince et al., 2017) such as non-reducing soluble sugars, raffinose family oligosaccharides (RFO proteins) (Salvi et al., 2016), LEAs and HSPs (Tejedor-Cano et al., 2010; Chatelain et al., 2012) act as chaperones and molecular shields in preventing protein denaturation and membrane destabilization during drying (**Figure 2**). Induction of Mt-HSFA9 expression is also correlated with the acquisition of longevity (Lima et al., 2017). The expression profiles of twenty-seven transcription factors highly correlated with longevity, including homologues of ERF110, HSF6AB, NFXL1, and members of the DREB2 family, as well as those associated with auxin (Arif et al., 2022).

### 4.3 Detoxification

#### 4.3.1 ROS scavenging

In order to control free radical induced cellular damage, seeds develop a detoxification mechanism that includes a number of antioxidant enzymes such as superoxide dismutase (SOD), catalase (CAT), ascorbate peroxidase (APX), mono-dehydroascorbate reductase (MDHAR), dehydroascorbate reductase (DHAR), glutathione peroxidase (GSHPx), and glutathione reductase (GSSGR) (Bailly, 2004; Bailly et al., 2008). A large number of these enzymes involved in ROS detoxification are present in dry mature seeds and germinating seeds. The compounds like glutathione, tocopherols, flavanoids and lipocalins limit oxidation of proteins, fats and nucleic acids (Lima et al., 2017). Scavenging of ROS is also known to be undertaken by glutathione, ascorbic acid (vitamin C) and peroxiredoxins.

## 5 Genetics of seed longevity

In soybean, a strong maternal influence is observed for seed coat characteristics and longevity of F_1_ seeds in reciprocal crosses (Kueneman, 1983) in addition to a minor influence of the seed’s own genotype. The segregation of F_2_ population in 3:1 and backcross population in 1:1 ratio confirms single gene control of seed longevity in soybean (Adsul et al., 2018). Seed longevity is also strongly associated with number of pods per plant and seed yield (Singh et al., 2021). The backcross/pedigree method of breeding is a better approach for transfer of seed storability trait to high yielding genotypes, however selection should not be practiced at F_2_ generation because trait expression is delayed by one generation during segregation due to strong maternal influence (Singh and Ram, 1986). The molecular and morphological distance-based grouping and model-based structuring of 96 soybean genotypes grouped six black seed coat colour genotypes viz., local black soybean, Kalitur, ACC Nos. 39, 109, 101 and 37 with high seed longevity and small seed size into one cluster (Naflath et al., 2022).

Stable genomic regions governing the inheritance of soybean seed storability comprising of 34 QTLs on 11 chromosomes were identified by employing high-density genetic linkage maps for seed longevity in two RIL populations derived from Zhengyanghuangdou Meng 8206 and Linhefenqingdou Meng 8206 (Zhang et al., 2019). Two major QTLs and eight QTL hotspots localized on chromosomes 3, 6, 9, 11, 15, 16, 17, and 19 are responsible for seed vigor related traits in soybean (Wang et al., 2021). Gene editing using CRISPAR cas9 for storage protein *viz*., Glyma.20g148400, Glyma.03g163500 Glyma.19g164900 was identified in soybean (Li et al., 2019). The candidate genes controlling nodulation specificity have been edited by CRISPR/Cas9 system combined with hairy-root transformation system (Tang et al., 2016). In 2014, TALENs technology was first used in soybean to mutate two fatty acid desaturase 2 genes (FAD2-1A and FAD2-1B) to create a high oleic acid soybean variety (Haun et al., 2014).

## 6 Molecular mechanism of seed longevity

Four independent SSR markers *viz., Satt538, Satt600, Satt434* and *Satt285* located at a distance of 158.63 cM, 75.4 cM, 105 cM and 25.51 cM on chromosomes A2, D1b, H and J respectively are associated with seed longevity (Cregan et al., 1999); *Satt434, Satt538, Satt281*, and *Satt598* on chromosomes H, A2, C2 and E respectively with seed coat permeability while *Satt28* on chromosome C2 is linked to electrolyte leaching (Singh et al., 2008b). The black and yellow seed coat genotypes were grouped by SSRs into two major clusters with *Satt371, Satt453* and *Satt618* that are identified as candidate markers linked with seed storability and testa color (Hosamani et al., 2013). Twenty seven transcription factors highly correlated with longevity *i.e*., homologues of *ERF110*, *HSF6AB*, *NFXL1* and members of the *DREB2* family based on gene co-expression network analysis. A transcriptional transition enriched with AP2/EREBP and WRKY transcription factors and genes associated with growth, germination and post-transcriptional processes occurred concomitant with seed chlorophyll loss and detachment from the mother plant, suggesting that this prepares the seed for the dry quiescent state and germination (Lima et al., 2017). Complex alterations occur in transcriptomes from seed physiological maturity till dry state indicating the role of transcription factors in delaying seed deterioration (Ramtekey et al., 2022). Metaanalysis and transcriptome profiling identified involvement of 7 hub genes in seed oil and seed storage protein accumulation processes in soybean (Qi et al., 2018).

Five QTLs located in chromosomes 2, 6 and 8 were found to be associated with seed viability and seed vigor and two QTLs for seed storability in soybean. These QTLs were found near loci controlling seed viability, maturity, germination, seed hardness, and seed surface micromorphology (Jiamtae et al., 2022). The genes linked to seed longevity in soybean include *GolS1_A*, *GolS2_B* and *RS2_B* associated with the synthesis of RFO (Le et al., 2020; de Koning et al., 2021), *RS1, RS2* and *RS3* involved in synthesis of raffinose (Dierking and Bilyeu, 2008), *SS* involved in synthesis of stachyose (Qiu et al., 2015) and *RS2* for synthesis of raffinose and stachyose (Valentine et al., 2017). In pea, soybean and chickpea, RFO levels and expression of genes regulating its biosynthesis such as ABI5, raffinose synthase and galactinol synthase control seed longevity and vigour during seed maturation and germination (Salvi et al., 2016; Zinsmeister et al., 2016; Lima et al., 2017). RFOs enhance seed vigour and longevity by repressing ROS production and lipid peroxidation (Salvi et al., 2016).

## 7 Post harvest measures to maintain seed longevity

Appropriate scheduling of harvest time is pivotal for seed quality as early harvest dampens seed longevity owing to under development of vital seed organs while delayed harvest delivers aged seed besides conferring losses due to seed shattering. Soybean seed should be harvested and threshed when seed moisture content is between 15 to 17%. Harvesting of soybean between yellow and brown-pod stages followed by drying within pods at low temperatures are optimal conditions for maintaining high seed quality (Ennen, 2011). During mechanical threshing soybean with weak structure is subjected to thrust leading to seed deterioration (Gagare et al., 2014; Kuchlan et al., 2018). High quality soybean seed with minimal damage is obtained at 14.3% moisture content in an axial flow thresher with a feed rate and cylinder speed of 600 to 700 rpm (13.2 to 15.4 m/s) and 720 kg (plant)/h respectively (Vejasit and Salokhe, 2004). During processing, static batch drying at temperatures upto 40°C, employing low-moving equipment and abrupt contacts with mechanical systems like pneumatic and gravity separators is recommended to minimize dropping and contact with seeds (Jaques et al., 2022).

Harvested seed should be stored at a seed moisture content of 8 to 12% based on the storage containers used. Processing of soybean seed in air-screen cleaner followed by gravity separator without a vertical bucket elevator for handling, improved the apparent seed germination by removing damaged seeds (Pardea et al., 2002). Enough care should be taken to ensure that the seed moisture content does not drop below 5%, else membrane structures may break down hastening the seed deterioration process. Seed longevity is greatly influenced by external storage conditions like temperature and humidity (Nadarajan et al., 2023) as well as diseases and insect pests. Soybean seed stored in polyethylene-coated raffia packaging under ambient conditions had physical and physiological qualities similar to those stored under refrigeration (Coradi et al., 2020). Treated soybean seeds remain viable for two seasons given the storage temperature is maintained at 10 °C and relative humidity is below 40% (Mbofung et al., 2013). Seed stored at relative humidity of 50 to 60% in polythene bags at an initial moisture content of 8% retained 80% germination up to six months (Ali et al., 2014).

## 8 Seed longevity Status of Indian soybean varieties

A vast number of soybean varieties (around 97) were developed and so far released in India since its introduction in 1970s. These varieties were grouped based on seed longevity and field weathering (Bhatia et al., 2006) in the order of highly tolerant (less than 15%), moderately tolerant (16 to 30%), susceptible (31 to 45%) and highly susceptible (more than 45%). It is worrying to note that only eight varieties were highly tolerant (Punjab 1, Kalitur, JS 80-21, NRC 37, JS 72-280, Lee, PK 471 and MACS 450), 34% were moderately tolerant (eg., JS 90-41, PK 472, MACS 124, and PK 1024) while major chunk fell in susceptible and highly susceptible classes. Several varietal characters like seed size, days to maturity, percentage of hard seeds, seed coat thickness, hull percentage, oil content, *etc*., contribute to the genotypic variation for field weathering (Bhatia, 1996; Bhatia et al., 1996; Singh et al., 2008a). In general early maturing varieties with bold seed, thinner seed coat, high seed coat permeability, fewer hard seeds and high oil content tend to lose viability much faster (Tiwari and Bhatia, 1995; Bhatia, 1996); while those with smaller sized seeds (Hosamani et al., 2013) and black seed coats (Kuchlan et al., 2010; Pawar et al., 2017; Adsul et al., 2018; Chandra et al., 2021) possess higher longevity.

## 9 Conclusion

Seed longevity is a major concern in soybean especially in tropical regions where retention of seed germination during the succeeding seasons is highly uncertain. Seed viability gradually declines during storage, and the extent of deterioration depends on storage conditions, nature and degree of damage incurred during post harvest operations and genetic architecture of the seed itself. Seed longevity in soybean is a complex phenomenon governed by the seed coat structure, membrane permeability, antioxidant system, genetic makeup and underlying molecular mechanisms. High lignin content in seed coat, glassy state of cells, improved antioxidant system and surplus storage proteins in seed form the cues for extension of seed longevity along with introgression of appropriate QTLs for delaying seed deterioration. Application of suitable nutrient and antioxidants to improve seed coat integrity and antioxidant system is another possible avenue. Nonetheless, further studies are needed for exploration and identification of suitable alternate niches for quality seed production of soybean in tropics. Given the growing preference of the crop and gravity of the persevering challenges involved, it is pertinent to understand the intricacies of molecular and genetic bases of mechanisms conferring seed longevity for formulation of breeding programmes integrated with marker assisted selection to derive lines with improved seed longevity in soybean.

## Author contributions

PR, MP, YB and PP: Writing the original draft and review. MP and YB: Writing review. PR: Review, supervision. PR, MP, YB, PP, PS and KP: Editing and visualization. All authors contributed to the article and approved the submitted version.
